# Comparative evaluation of caries detector dyes and laser fluorescence systems for intraoperative diagnosis during selective caries removal: a scoping review

**DOI:** 10.3389/fdmed.2025.1600500

**Published:** 2025-07-16

**Authors:** Ana Iglesias-Poveda, Javier Flores-Fraile, Diego González-Gil, Joaquín López-Marcos

**Affiliations:** Surgery Department, Dental Clinic Faculty of Medicine, University of Salamanca, Salamanca, Spain

**Keywords:** selective caries removal, intraoperative caries assessment, caries detector dyes, laser fluorescence systems, magnification, pulp vitality, minimally invasive dentistry

## Abstract

**Background:**

Selective caries removal aims to preserve pulp vitality and tooth structure by eliminating only infected dentin. Caries detector dyes and laser fluorescence devices are the main diagnostic tools supporting this minimally invasive approach.

**Objective:**

To evaluate and compare the diagnostic performance, benefits, and limitations of these two modalities. Additionally, it examines potential synergies with magnification tools and proposes future directions for clinical protocol development.

**Methods:**

A scoping review was conducted following PRISMA-ScR guidelines. Of 124 articles screened, four met the inclusion criteria for direct comparison of caries detector dyes and laser fluorescence systems. Diagnostic accuracy, clinical outcomes, and bias risk (ROBINS-I/ROBINS-E) were assessed.

**Results:**

All four studies supported the effectiveness of both techniques in selective caries removal. Laser fluorescence devices showed higher sensitivity (ranging from 0.76 to 0.92) and specificity (0.74 to 0.88), along with better accuracy in detecting infected dentin compared to dyes. Dyes were noted for ease of use but showed greater variability in outcomes. Risk of bias ranged from low to moderate across studies.

**Conclusions:**

Laser fluorescence systems appear to be more reliable for intraoperative caries detection during selective caries removal. Nevertheless, a combined diagnostic approach, particularly with magnification, may optimize outcomes. These findings support the integration of fluorescence systems in caries management protocols. Further clinical trials are needed to validate these findings and develop standardized, evidence-based protocols.

## 1 Introduction

Dental caries is a chronic, multifactorial disease influenced by biological, behavioural, and environmental factors (1). It results from an imbalance between demineralization and remineralization processes mediated by biofilm activity (2). Accurate detection and proper management of carious lesions are essential to prevent disease progression, preserve tooth structure, and maintain pulp vitality (3).

Minimally invasive dentistry has gained prominence in recent years, promoting conservative strategies that prioritize tissue preservation and long-term tooth survival (4). In this context, visual-tactile inspection remains the most widely used diagnostic method. Systems such as the International Caries Detection and Assessment System (ICDAS) provide a standardized and reproducible approach to assess lesion activity and depth (5). Despite its reliability, the accuracy of visual inspection can be affected by clinical conditions and examiner experience (6).

To enhance diagnostic precision, adjunctive technologies have been developed. Fluorescence-based devices, such as DIAGNOdent and QLF, detect variations in fluorescence that correspond to demineralized tooth structure or bacterial byproducts (7). These tools have shown promise, particularly in early lesion detection, but their sensitivity and specificity vary across clinical studies (8, 9).

In cases of deep carious lesions, accurate diagnosis is particularly important, as it determines the choice of appropriate minimally invasive approaches, such as selective or stepwise caries removal, which aim to preserve pulp vitality and avoid unnecessary tissue removal. These techniques offer a conservative alternative to non-selective caries removal, which increases the risk of pulpal exposure and irreversible damage (10–14).

Previous studies have shown that selective carious tissue removal offers several advantages, including reduced risk of pulp exposure, preservation of dental and pulp tissues, and greater restoration longevity (15–17). These benefits have positioned it as one of the most recommended operative procedures in current conservative dentistry. Clinically, caries evaluation often relies on the colour and hardness of dentin. However, this method is subjective and has low reproducibility (18, 19). To improve accuracy, caries detector dyes were introduced. These dyes aim to highlight infected dentin while preserving healthy tissue (5, 20).

In 1972, a technique using a basic fuchsin red stain was suggested (and subsequently developed) to aid in the differentiation of the two layers of carious dentin (5, 6). Because of potential carcinogenicity, the basic fuchsin stain was subsequently replaced by another dye, acid red solution (7). Since then, various protein dyes have been marketed as caries detection agents (21). Caries detector dyes typically contain 1% acid red in propylene glycol and help clinicians distinguish demineralized dentin from sound tissue during excavation (22). Nevertheless, studies have reported that these dyes can overstain and lead to the unnecessary removal of sound dentin (5, 18, 23). Conversely, other investigations suggest that these dyes can aid detection, and their absence might result in undiagnosed carious tissue (19, 24–27).

In addition to chemical agents, fluorescence-based devices have been introduced. The DIAGNOdent (KaVo Dental, Biberach, Germany) uses laser fluorescence (wavelength 655 nm) to detect caries by measuring fluorescence emitted by bacterial porphyrins (28, 29). This device quantifies lesion severity numerically, offering an objective diagnostic tool (30–33).

Additionally, the Fluorescence-Assisted Caries Excavation (FACE) system (Selbekk, Oslo, Norway) enables clinicians to identify infected dentin by directly observing red-orange fluorescence, avoiding the use of dyes (34, 35). The DIAGNOdent Cam, functioning similarly to Quantitative Light-induced Fluorescence (QLF), also relies on fluorescence emissions to detect early demineralization, highlighting a diagnostic overlap that warrants further comparative studies.

To improve the sensitivity and reliability of caries detection methods, it is suggested to correlate visual and tactile criteria with microbial activity in dentin. This approach could provide a more accurate and reproducible method for both quantitative and qualitative assessment (36–38).

Therefore, this scoping review aims to evaluate diagnostic methods that support effective selective caries removal. Specifically, it compares the clinical performance of caries detector dyes and laser fluorescence devices in terms of diagnostic accuracy and support for minimally invasive approaches, such as stepwise and selective removal techniques. The review also explores their advantages regarding dental tissue preservation, pulp vitality maintenance, and the long-term success of restorative treatments.

## 2 Material and methods

### 2.1 Study design

This scoping review was conducted in accordance with the PRISMA-ScR guidelines. A completed PRISMA-ScR Checklist is provided as Supplementary Material to ensure transparency and adherence to established reporting standards.

We A bibliographic search was carried out using the PubMed/MEDLINE and Web of Science databases to collect articles published over the last 15 years (from January 2008 to December 2024, an adequate timeframe to assess potential advancements in the management of carious lesions, including only articles published in English.

The PICO strategy (11). Population (P): Patients with permanent teeth requiring selective caries removal. Intervention (I): Use of caries detector dyes during selective caries removal. Comparison (C): Laser fluorescence devices (e.g., DIAGNOdent) or conventional visual/tactile examination.

Outcome (O): Diagnostic effectiveness (sensitivity, specificity, accuracy) and clinical impact (dentin preservation, pulp vitality preservation).

Minor adjustments to the initial research strategy were made to include studies comparing dyes with either laser fluorescence or conventional visual/tactile assessments, to ensure a comprehensive evaluation of diagnostic methods.

To ensure relevant studies were identified, this review focused on permanent dentition due to key anatomical and histological differences that limit the applicability of findings from primary teeth. Thinner enamel, greater permeability, and distinct pulp responses in primary teeth influence caries progression and treatment outcomes. Since permanent teeth require long-term restorative strategies and pulp vitality preservation, this study aims to establish clinically relevant guidelines.

Thus, this systematic review included meta-analyses and RCTs addressing the question: “*In permanent teeth with deep lesions, which diagnostic method (staining techniques and/or conventional radiographs or laser fluorescence) provides the best clinical outcome and success?”*

The search strategy was specifically developed for each database. The protocol was registered in the International Prospective Register of Systematic Reviews (PROSPERO, http://www.crd.york.ac.uk/prospero) under registration number CRD42024608203.

### 2.2 Inclusion criteria

Due to the limitations in the number of articles found in the scientific literature addressing this topic, we only included meta-analyses comparing conventional and current techniques for the diagnosis and removal of caries in permanent teeth of healthy patients, as well as articles on cavitated dentin lesions. Scientific articles written in English and Spanish with full text available were also included.

### 2.3 Exclusion criteria

**Figure 1:**
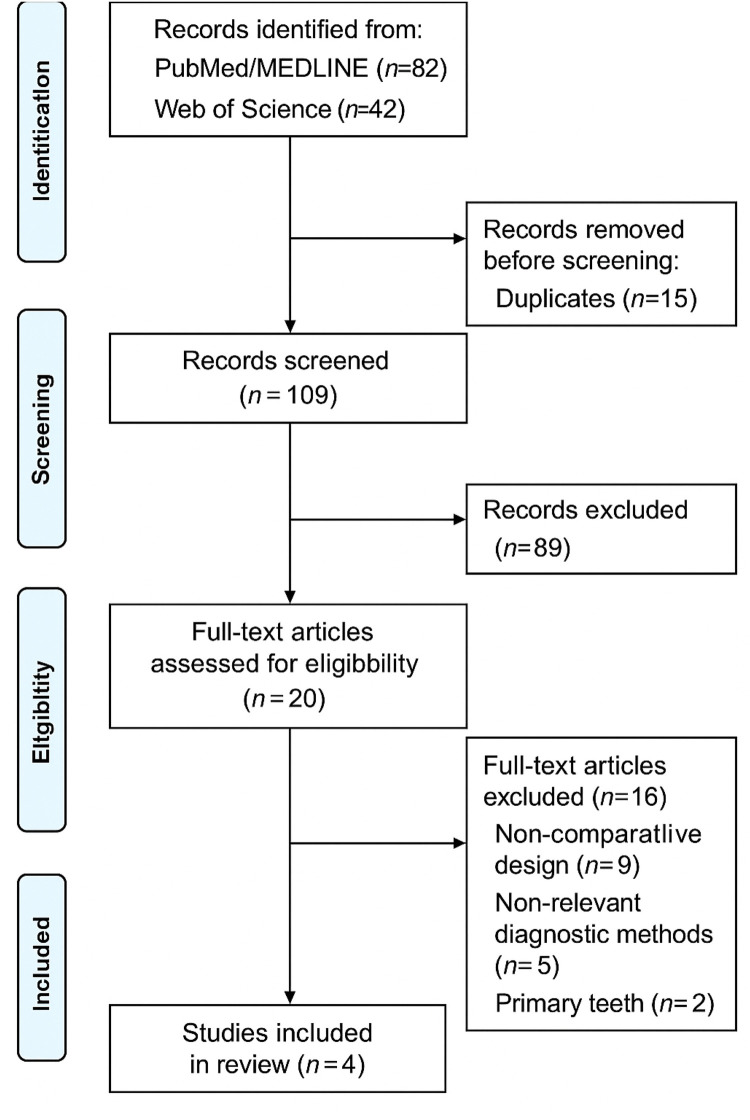
PRISMA flow diagram.

After conducting the scoping review and screening the titles and abstracts of the selected studies, we observed that most of the research focused on primary dentition. Consequently, we excluded all articles examining techniques in primary dentition, as well as unfinished trials or studies conducted in specific populations (special care needs, infectious diseases, or syndromic patients). This process is illustrated in the flow diagram (Figure 1).

### 2.4 Quality assessment of included studies

The risk of bias of non-randomized studies was assessed using the ROBINS-I tool, while randomized controlled trials were evaluated with the ROBINS-E tool. For each domain of the respective tools, studies were rated as “Low risk,” “Some concerns,” or “High risk” based on predefined signaling questions.

A study was classified as “Low risk” if no significant sources of bias were identified across all domains. “Some concerns” were assigned when minor issues were present without significantly affecting overall validity. “High risk” was assigned if major methodological flaws were found that could substantially impact the study's results.

Risk of bias assessment was conducted independently by two reviewers, and any discrepancies were resolved through discussion.

This tool is structured into five domains (D) where potential bias can be assessed. And we evaluated one study using the ROBINS-E toll (Risk Of Bias In Randonmized Studies) designed to assess the risk of bias in ECAS. It provides signaling questions whose answers indicate the potential for bias, offering a systematic way to organize and present the available evidence related to the risk of bias in NRS.

### 2.5 Variables

The following variables were predefined for extraction to systematically characterize and compare the included studies:

• Diagnostic method evaluated: Identification of whether the study assessed caries detector dyes, laser fluorescence devices (e.g., DIAGNOdent), or fluorescence-aided caries excavation (FACE) techniques.
• Comparator method: Determination of the reference method used for comparison, including visual-tactile inspection or traditional caries detection techniques.
• Study design: Classification of the methodological design as clinical trial, observational study, or randomized controlled trial (RCT).
• Diagnostic performance outcomes: Extraction of quantitative data such as sensitivity, specificity, positive predictive value (PPV), negative predictive value (NPV), and overall diagnostic accuracy for each method evaluated.

In studies involving laser fluorescence systems such as DIAGNOdent, the diagnostic threshold values used to interpret fluorescence readings were also considered to ensure consistent data extraction.

Typically, readings above 30 were interpreted as indicating carious dentin requiring removal, values between 14 and 30 were associated with enamel demineralization, and values below 13 represented sound, healthy tissue.

• Clinical impact outcomes: Reporting of any assessments related to dentin preservation, pulp vitality maintenance, or avoidance of pulp exposure during selective caries removal.
• Risk of bias assessment: Documentation of the risk of bias evaluation using the ROBINS-I tool for non-randomized studies and the ROBINS-E tool for RCTs, including domain-specific assessments.

These variables were selected to comprehensively evaluate both the diagnostic efficacy and clinical relevance of different caries detection methods during selective caries removal procedures.

### 2.6 Research strategy

To identify studies for this review, scientific literature databases such as Medline/PubMed (http://www.ncbi.nlm.nih.gov/pubmed). The search strategy was first developed for Medline/PubMed using a combination of controlled vocabulary and free-text terms and then appropriately adapted for each database.

The following terms were used, both as free-text words and, where applicable, as Medical Subject Headings (MeSH) or equivalent thesaurus terms: “selective caries removal”, “laser fluorescence” and “caries detector dyes.” A sensitive filter was created by combining the three filters for identifying diagnostic studies using the Boolean operators “OR” and “AND.” Additionally, the social network ResearchGate was used to obtain fulltext articles with the consent of their authors.

Minor deviations from the original PROSPERO protocol (CRD42024608203) were introduced to optimize the sensitivity and specificity of the literature search. Specifically, slight adjustments were made to the search terms and database selection to ensure a more comprehensive coverage of the relevant scientific evidence. These modifications were performed prior to study selection and data extraction, maintaining the methodological integrity and objectives initially established in the protocol.

## 3 Results

The initial database search yielded a total of 124 records (PubMed/MEDLINE: 82; Web of Science: 42). After removal of 15 duplicates, 109 titles and abstracts were screened for eligibility. Following the screening process, 89 records were excluded based on title and abstract review for reasons such as irrelevant outcomes, study design not meeting inclusion criteria, or evaluation of non-targeted diagnostic methods.

A total of 20 full-text articles were assessed for eligibility. Of these, 16 studies were excluded for the following reasons: non-comparative design (*n* = 9), use of different diagnostic tools unrelated to caries detector dyes or laser fluorescence (*n* = 5), and studies involving primary teeth (*n* = 2). Ultimately, 4 studies were included in the final qualitative analysis.

The study selection process is illustrated in Figure 1.

Study designs: The selected studies (39–42) conducted between 2015 and 2024. These studies are shown in Table 1. Additionally, there was a lack of homogeneity in conventional and minimally invasive methods, requiring the combination of similar procedures with variations in their protocols.

**Table 1:** Characteristics of the included studies.

Study	Year	Study desing	Diagnostic method evaluated	Comparison method
Peskersoy et al	2015	Clinical Trial	Fluorescence Aided Caries Excavation (FACE)	Caries Detector Dyes (CDD)
Koç Vural et al.	2017	Observational study	FACE	Visual-Tactile Method
Sadavisa et al.	2019	*in vivo* study	DIAGNOdent (KaVo Dental)	CDD + Visual-Tactile Method
Abba et al.	2024	Randomized controlled trial	DIAGNOdent (KaVo Dental)	CDD + Visual-Tactile Method + PCR Bacterial Analysis

### 3.1 Risk of bias assessment

Risk of bias assessment was performed for all included studies using the ROBINS-I tool for non-randomized studies and the ROBINS-E tool for the randomized controlled trial. Among the four studies, two were judged to have a low risk of bias, one study presented some concerns, and one was classified as high risk.

**Figure 2:**
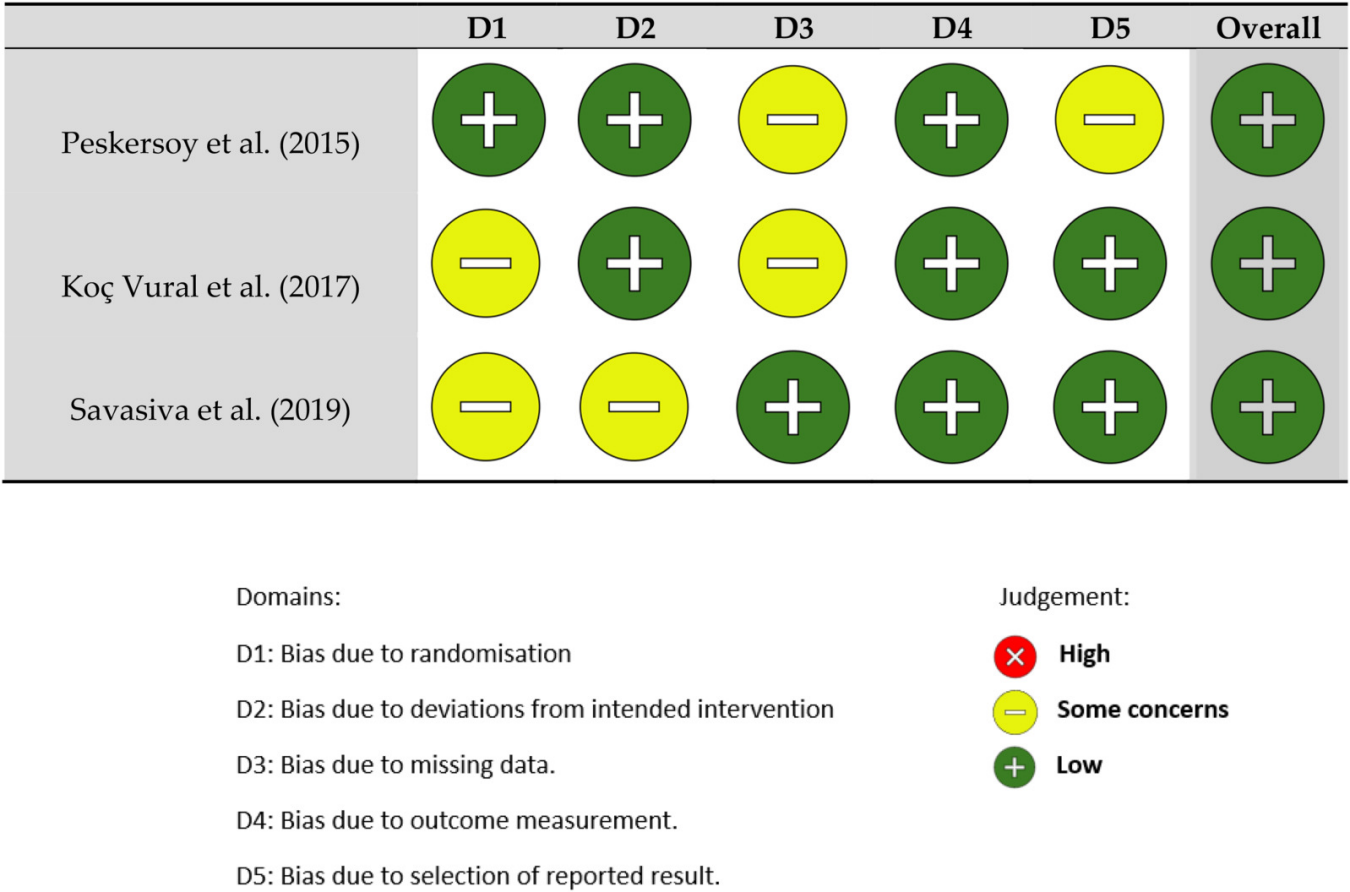
ROBINS-I tool (risk of bias in non-randomized studies).

**Figure 3:**
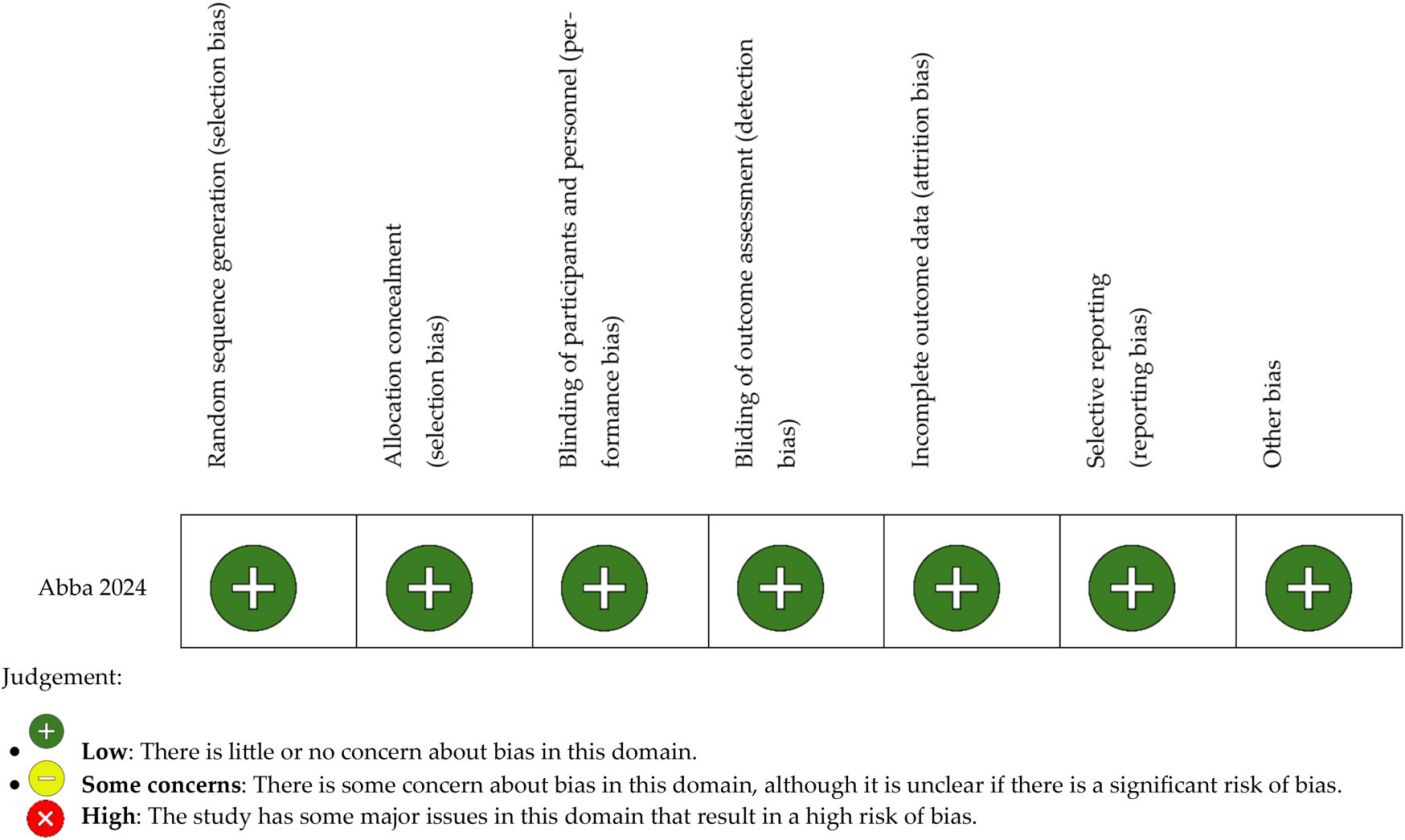
ROBINS-E bias tool (risk of bias in randomized studies).

The main sources of bias identified were related to potential selection bias, inadequate blinding during outcome assessment, and reporting bias. A detailed overview of the risk of bias assessments is presented in Figure 2 (ROBINS-I) and Figure 3 (ROBINS-E).

**Figure 4:**
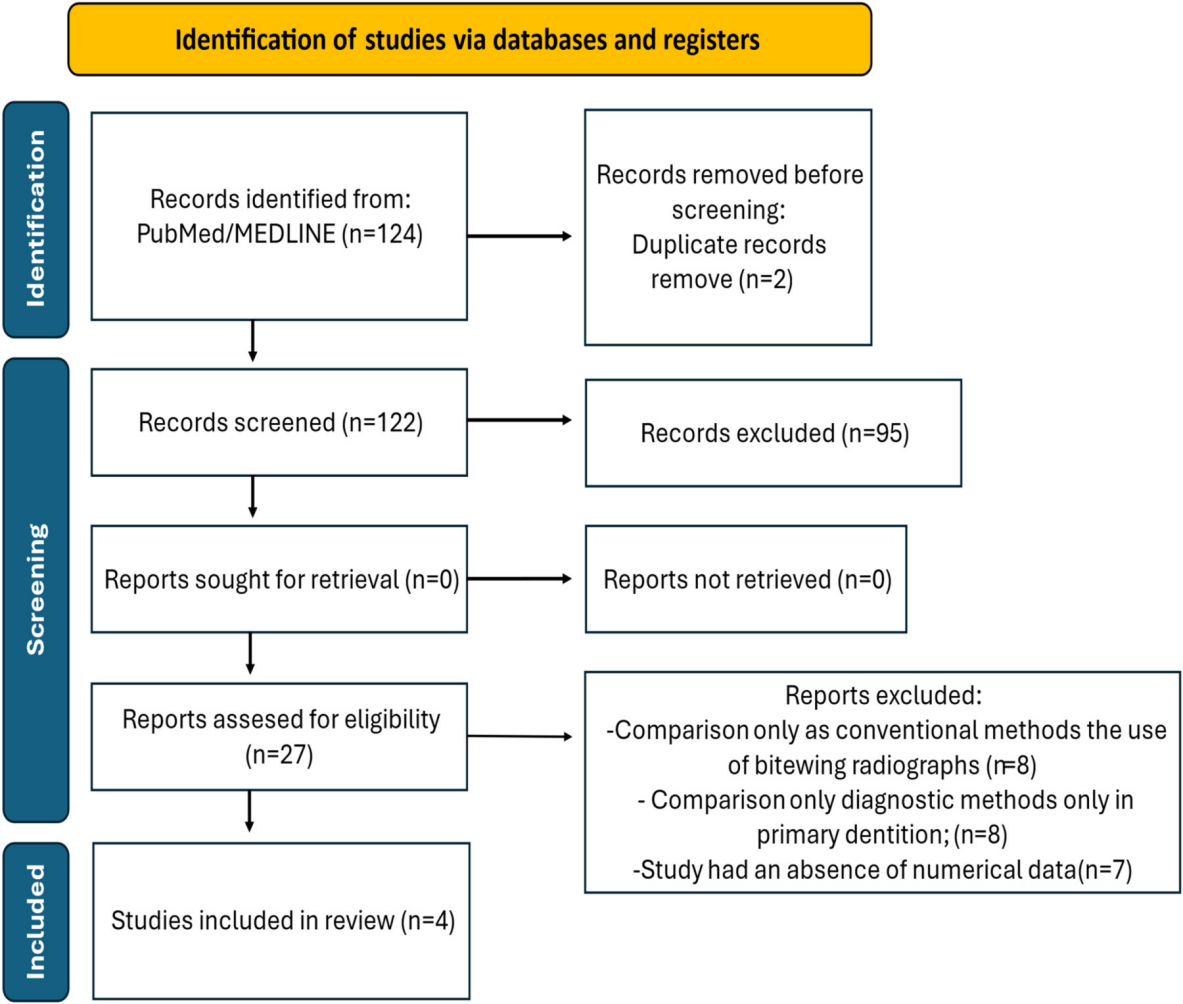
Flowchart showing systematic review.

The flow diagram illustrating the study selection process is shown in Figure 4.

The findings in Figure 2 highlight key differences in caries detection methods. Peskersoy et al. (39) and Koç Vural et al. (40) showed that fluorescence-assisted excavation (FACE) significantly reduces residual caries compared to conventional methods. Sadasiva et al. (41) found that combining caries detector dyes with laser fluorescence enhances diagnostic accuracy and minimizes unnecessary dentin removal. Abba et al. (42) further confirmed that DIAGNOdent achieves the highest sensitivity and specificity, making it a valuable tool for selective caries removal. Similarly, Koç Vural et al. (40) reinforced this perspective by comparing different detection techniques and their impact on clinical decision-making. Their study assessed the diagnostic accuracy of visual, radiographic, and staining methods in determining the extent of dentin removal.

### 3.2 Diagnostic performance

All included studies reported diagnostic performance data for the evaluated methods. Laser fluorescence devices consistently demonstrated higher sensitivity and specificity values compared to caries detector dyes. Sadasiva et al. (41) and Koç Vural et al. (40) showed that fluorescence-based detection methods achieved superior diagnostic accuracy compared to visual-tactile inspection alone. Meanwhile, caries detector dyes, although effective in identifying infected dentin, exhibited lower specificity and a tendency towards over-excavation. The diagnostic performance metrics for each study are presented individually in Table 2.

**Table 2:** Summary of included studies comparing diagnostic techniques.

Title	Author and year	Clinical question	Type of technique	Results
Comparative clinical evaluation of the efficacy of a new method for caries diagnosis and excavation.	Peskersoy et al. 2015 (39)	To evaluate the null hypothesis that FACE is an ad-vanced diagnostic method compared to caries detector dyes (CDD) and visual in-spection, the following steps can be undertaken.	The results of this study demonstrated that the incidence of residual caries after evaluation with FACE was significantly lower than that of conventional visual evaluation and the application of a caries detector dye.	The findings of this study reveal that FACE is an effective, clinically applicable, and straightforward method for diagnosing sound and carious dentin.
Comparison of two different methods of detecting residual caries.	Koç Vural et al. 2017 (40)	The aim of this study was to investigate the ability of the fluorescence-aided caries ex-cavation (FACE) device to detect residual caries by comparing conventional methods *in vivo*.	Significant differences were found between visual inspection with or without magnifying glasses and inspection with a FACE device for all groups (*p* < 0.001). More residual caries were detected through inspection with a FACE device (46.5%) than through either visual inspection (31.8%) or inspection with a magnifying glass (37.6%).	While additional research is necessary, the encouraging findings from this study indicate that fluorescence-assisted operative techniques (FACE) could enhance the effectiveness of caries removal and the identification of remaining caries during cavity preparation.
Evaluation of the Efficacy of Visual, Tactile Method, Caries Detector Dye, and Laser Fluorescence in Removal of Dental Caries and Confirmation by Culture and Polymerase Chain Re-action: An *in vivo* Study.	Sadasiva et al. 2019 (41)	The aim was to determine the degree of association between visual and tactile methods of caries removal compared with caries detector dye and laser fluorescence device (DIAGNOdent), which detects the degree of demineralization and to find a suitable method for caries removal.	Pearson's chi-square test was conducted to compare the PCR and culture values in all the three groups, and the results were found to be statistically significant. The combination of visual and tactile examination, caries detector dye, and laser fluorescence (Group C) was found to be the most effective method for caries removal.	Incorporating caries detector dye and laser fluorescence with traditional visual and tactile methods enhances the efficiency of caries removal, aiding clinicians in preserving tooth structure while ensuring thorough debridement.
Evaluation of residual carious dentin detection methods after cavity preparation: a randomized clinical trial.	Abba et al. 2024 (42)	This study aimed to determine the efficiency of visual-tactile, caries detector dye (CDD), and laser fluorescence (LF) device methods for diagnosing residual caries after cavity preparation.	The visual-tactile method had a specificity of 100%, CDD had 100% sensitivity and 92.9% specificity, and DIAGNOdent had 100% sensitivity and 100% specificity when measured against bacterial cultures. The positive predictive value for CDD (50%) was half that for DI-AGNOdent (100%). The accuracy was highest (100%) for DIAGNOdent.	DIAGNOdent was the most specific of the tested modali-ties and had the highest level of agreement with bacteriological confirmatory tests.

### 3.3 Clinical outcomes: tissue preservation, pulp vitality, and long-term results

Three of the included studies reported data specifically addressing clinical outcomes related to tissue preservation and pulp vitality during selective caries removal.

Abba et al. (42) demonstrated that the use of fluorescence-assisted detection significantly reduced the incidence of pulp exposure compared to conventional staining techniques. Their findings emphasized the importance of preserving affected but remineralizable dentin by improving intraoperative caries discrimination, enhancing restoration margins and reducing operative trauma.

Similarly, Sadasiva et al. (41) reported that fluorescence-guided caries removal allowed for a more conservative excavation approach, resulting in a greater amount of healthy dentin preserved without compromising caries removal efficacy. Their study highlighted the value of adjunctive fluorescence devices in maintaining pulp vitality during deep carious lesion management.

Koç Vural et al. (40) corroborated these findings, highlighting that fluorescence–based methods resulted in less invasive dentin removal compared to both traditional visual–tactile examination and caries detector dyes alone, despite not directly assessing pulpal outcomes. A detailed summary of these results is presented in Table 3.

**Table 3:** Comparative diagnostic performance of caries detector dyes vs. Laser Fluorescence Devices.

Diagnostic method	Sensitivity (range)	Specificity (range)	PPV (Approx.)	NPV (Approx.)	Diagnostic accuracy	Limitations
Caries detector dyes	0.60–0.80	0.50–0.75	0.58–0.70	0.72–0.85	Moderate	Potential overstaining of healthy dentin
Laser fluorescence devices	0.76–0.92	0.74–0.88	0.75–0.90	0.80–0.93	High	Cost, device calibration needed

Values reflect diagnostic performance ranges reported in the included studies.

Regarding long-term outcomes, Kanar et al. (2024) provided medium-term follow-up data suggesting that teeth treated with fluorescence-guided caries removal exhibited favorable restoration survival rates and a lower incidence of secondary caries compared to traditional diagnostic approaches. However, the study acknowledged limitations due to patient attrition and lack of control over oral hygiene behaviors during follow-up.

Collectively, these findings suggest that fluorescence-based diagnostic methods not only enhance immediate diagnostic accuracy but may also contribute to improved clinical outcomes through greater tissue preservation and pulp vitality maintenance. Nevertheless, high-quality, long-term randomized studies are necessary to substantiate these associations and guide clinical decision-making.

## 4 Discussion

Given the small number of studies directly comparing caries detector dyes and laser fluorescence methods, a descriptive comparison of the diagnostic values was performed. This allowed for a clearer interpretation of the diagnostic effectiveness of both methods, while respecting the methodological framework of a scoping review.

The findings in Figure 2 highlight key differences in caries detection methods. Peskersoy et al. (39) and Koç Vural et al. (40) showed that fluorescence-assisted excavation (FACE) significantly reduces residual caries compared to conventional methods. Sadasiva et al. (41) found that combining caries detector dyes with laser fluorescence enhances diagnostic accuracy and minimizes unnecessary dentin removal. Abba et al. (42) further confirmed that DIAGNOdent achieves the highest sensitivity and specificity, making it a valuable tool for selective caries removal. Similarly, Koç Vural et al. (40) reinforced this perspective by comparing different detection techniques and their impact on clinical decision-making. Their study assessed the diagnostic accuracy of visual, radiographic, and staining methods in determining the extent of dentin removal. The authors concluded that fluorescence-assisted excavation significantly enhances the identification of carious dentin while preserving as much healthy tissue as possible. On the other hand, Kanar et al. (2024) introduced a technological dimension with the use of laser fluorescence (LF) in evaluating caries removal techniques. Their study compared the effectiveness of DIAGNOdent and FACE with conventional methods, finding that fluorescence-based systems offer superior sensitivity and specificity. However, the authors noted that factors such as extrinsic pigmentation, tooth hydration, and detection threshold variability can influence their performance.

This scoping review identified that laser fluorescence-based diagnostic methods demonstrate higher sensitivity, specificity, and overall diagnostic accuracy compared to caries detector dyes in the context of selective caries removal. Both diagnostic approaches contribute to the clinical objective of preserving dentin and maintaining pulp vitality, aligning with the principles of minimally invasive dentistry. However, the available evidence remains limited, and further high-quality research is necessary to substantiate these findings and standardize clinical protocols. The primary aim of this review was to determine the most appropriate intraoperative diagnostic method to support selective caries removal by comparing the effectiveness of caries detector dyes and laser fluorescence systems in accurately identifying carious tissue, with a specific focus on tissue preservation, pulp vitality, and long-term restorative success.

A detailed comparative overview of the diagnostic performance, including sensitivity and specificity values across different studies and methods, is presented in Table 3. This table highlights the variability among techniques and supports the need for standardized protocols. Laser fluorescence systems demonstrate superior sensitivity, allowing for more reliable detection of residual infected dentin. This reduces the risk of undiagnosed carious tissue and helps ensure long-term restoration success. Similarly, the higher specificity of fluorescence-based methods minimizes the unnecessary removal of healthy, remineralizable dentin, reinforcing the goal of preserving pulp vitality and supporting the fundamental principles of selective caries removal. In contrast, the lower specificity associated with caries detector dyes may result in over-excavation and inadvertent pulp exposure, increasing the risk of operative complications and negatively impacting the long-term success of restorations. These differences in diagnostic performance have significant clinical implications, emphasizing the potential role of laser fluorescence systems as adjunctive tools to inform clinical decision-making in minimally invasive caries management. Fluorescence devices have demonstrated more consistent diagnostic performance across the analyzed studies, which may translate into greater clinical reliability. However, this hypothesis requires further validation through studies conducted in real-world clinical settings.

These findings underscore the clinical importance of selecting appropriate intraoperative diagnostic methods during selective caries removal. The greater diagnostic accuracy achieved with laser fluorescence systems—characterized by higher sensitivity and specificity—enables more reliable differentiation between infected and remineralizable dentin. This contributes to minimizing the risk of residual carious tissue and avoiding unnecessary dentin removal, both of which are critical to maintaining pulp vitality, especially in deep lesion management. Although current clinical outcome data are limited, existing evidence suggests that fluorescence-guided selective caries removal is associated with lower rates of pulp exposure and improved restorative prognosis compared to approaches relying solely on caries detector dyes. The integration of adjunctive diagnostic technologies within minimally invasive protocols may also support the standardization of clinical procedures, enhance pulp preservation, and reduce operative complications. Nonetheless, practical considerations such as the cost of devices, clinical accessibility, and the learning curve for clinicians must be addressed to facilitate the widespread adoption of these technologies in routine practice.

These practical barriers must be considered when evaluating the real-world applicability of these technologies. Although devices like DIAGNOdent and FACE offer high diagnostic accuracy and intraoperative utility, their broader clinical adoption is constrained by factors such as cost, the need for regular calibration, and operator training. DIAGNOdent is sensitive to external variables such as hydration or surface staining, which may affect its consistency. FACE, meanwhile, provides direct visual assessment of infected tissue but remains less accessible due to higher costs and limited availability. In contrast, caries detector dyes offer a low-cost, easy-to-use alternative, though their lower specificity can lead to overtreatment, particularly in the absence of magnification or standardized interpretation criteria. Therefore, selecting a diagnostic system should account not only for clinical efficacy but also for feasibility based on available resources, supporting a balanced integration into daily practice.

Despite the promising diagnostic performance of fluorescence-based methods, several limitations in the current evidence must be acknowledged. Studies such as Kanar et al. (2024) provide valuable medium-term data on restoration longevity and success but often face challenges related to patient attrition and the inability to control for confounding variables such as oral hygiene practices. Conversely, shorter-term studies (e.g., 40, 41) offer precise diagnostic performance data but do not assess the long-term clinical implications of these diagnostic methods. Furthermore, methodological heterogeneity among studies—in terms of excavation protocols, operator experience, and outcome measures—limits the generalizability of findings and hinders the establishment of definitive clinical guidelines.

To address these limitations, future research should focus on large-scale, randomized controlled trials with standardized diagnostic criteria, consistent operator training, and long-term follow-up. Patient-centered outcomes, including restoration survival, quality of life indicators, and cost-effectiveness, should be incorporated into study designs to enhance their clinical relevance. Moreover, the impact of factors such as the specific type of caries detector used, manual vs. rotary excavation techniques, and clinician expertise warrants further investigation to optimize selective caries removal strategies. The use of magnification tools, such as dental operating microscopes or magnifying loupes, may also contribute to greater precision by enabling the detection of minimally demineralized areas not visible to the naked eye. This could help reduce the risk of inadvertent pulp exposure or excessive removal of sound tissue. Additionally, conventional pulp sensitivity tests frequently employed in clinical settings do not reliably reflect the true inflammatory status of the pulp, further emphasizing the need for improved diagnostic strategies in the management of deep carious lesions.

Although this review has focused primarily on caries detector dyes and laser fluorescence systems, the potential synergy between these diagnostic tools and optical magnification warrants a more detailed discussion.

Koç Vural et al. (40) provided direct evidence supporting the added value of magnification. Their study compared visual inspection with and without magnifying glasses to fluorescence-aided caries excavation (FACE), showing that magnification significantly enhanced residual caries detection. Residual lesions were identified in 46.5% of cases using FACE, compared to only 31.8% with unaided visual inspection.

Despite this, most studies evaluating caries detector dyes do not specify whether magnification was employed during clinical assessment. For instance, Peskersoy et al. (39) and Sadasiva et al. (41) examined the efficacy of dyes and fluorescence systems but did not indicate the use of magnification, limiting our understanding of the dyes' full diagnostic potential. This methodological omission may result in an underestimation of the accuracy of dye-based diagnostics when used without enhanced visualization.

Moreover, evidence suggests that magnified visualization may not only complement but potentially rival laser fluorescence systems. Clinical scenarios have demonstrated that suspicious areas overlooked under normal vision become evident under dental operating microscopes, and when combined with dye application, allow for more targeted tissue removal. These findings highlight the diagnostic value of magnification-assisted techniques, particularly when paired with chemical indicators.

Further research is needed to specifically assess the performance of caries detector dyes under magnification and compare such combined strategies to advanced technologies like DIAGNOdent. Standardizing protocols that incorporate enhanced optical tools could improve the precision and consistency of selective caries removal, providing an effective and potentially more accessible alternative to fluorescence-based diagnostics.

From a therapeutic perspective, incorporating fluorescence-based methods could enhance the standardization of selective caries removal, reducing the risk of pulp exposure and unnecessary tissue loss. Given that permanent teeth require long-term restorative strategies, optimizing diagnostic precision is critical for ensuring restoration durability and pulp vitality. Although fluorescence-based techniques show promise in permanent dentition, further high-quality research is needed to develop robust clinical guidelines. Large-scale randomized controlled trials should evaluate their long-term efficacy, considering patient-specific variables and standardized protocols. Integrating these methods into clinical practice could improve diagnostic reliability and lead to more predictable treatment outcomes in restorative dentistry. Long-term follow-up studies, such as that by Kanar et al. (2024), provide valuable data on restorative longevity and medium-term outcomes but face limitations such as patient attrition and uncontrolled variables like oral hygiene habits. Conversely, studies with shorter follow-up periods, such as those by Sadasiva et al. (41) and Koç Vural et al. (40), yield immediate and specific results on diagnostic performance but do not adequately address long-term impact.

These factors influence the observed outcomes and should be considered in future investigations. While meta-analyses are critical for consolidating scientific evidence, the number of comparative studies retrieved in this review remains modest. Alternative methodological approaches could help fill existing gaps. Furthermore, variables related to caries removal procedures, including the type of detector used, excavation technique, and clinician experience may affect outcomes and should be systematically addressed in future reviews. Integrating advanced technologies such as laser fluorescence into clinical practice has the potential to significantly improve outcomes, particularly in cases where the preservation of healthy tissue is critical. Nonetheless, implementation must account for factors such as device cost, availability, and the associated learning curve. A combined diagnostic approach may offer a balanced solution, maximizing diagnostic efficacy while mitigating the limitations inherent to individual techniques.

Although studies conducted in primary dentition dominate the literature, available evidence suggests that fluorescence-based techniques and caries detector dyes are equally effective in permanent teeth. Given the structural and functional differences between primary and permanent dentition, it is essential to further investigate long-term outcomes in adult populations. Ensuring diagnostic accuracy in permanent teeth is crucial for maintaining pulp vitality and optimizing restorative success, reinforcing the need for continued clinical trials. The validation and integration of fluorescence-based methods into standardized clinical protocols is essential to enhance diagnostic reliability, optimize restorative outcomes, and ensure the longevity of restorations in permanent dentition.

In addition to reporting diagnostic performance, it is crucial to critically assess the practical limitations and clinical implications. Although laser fluorescence systems generally show superior sensitivity and specificity, their performance can be influenced by extrinsic staining, hydration levels, and variations in calibration, potentially leading to false positives or inconsistent readings. On the other hand, caries detector dyes, while simpler and more cost-effective, are prone to overstaining, which may result in overtreatment and unnecessary dentin removal—especially when used without magnification or objective thresholds. This comparative analysis highlights the trade-off between accessibility and diagnostic accuracy. The combined use of dyes and fluorescence has shown promise yet lacks standardized protocols and long-term clinical validation. Therefore, future directions should aim to refine existing methods by incorporating visual enhancement tools, improving diagnostic selectivity, and developing integrative approaches that overcome the individual weaknesses of each modality. Such critical evaluation not only enhances the scientific depth of this review but also provides clinicians with a more nuanced understanding of the tools available for minimally invasive caries management.

A combined approach utilizing fluorescence–based systems together with visual aids, such as magnification, may provide the most balanced strategy enhancing diagnostic accuracy while minimizing unnecessary dentin removal and overtreatment in deep carious lesions. Clinical decision–making protocols for selective caries removal in different scenarios are summarized in Table 4.

**Table 4:** Clinical decision protocol for caries removal.

Clinical scenario	Recommended diagnostic systems	Caries removal strategy	Notes
General assessment	Visual-tactile + ICDAS	Lesion classification	Use magnification where possible
Moderate lesion	Caries detector dye (CDD)	Selective removal	Simple; may overstain healthy dentin
Deep lesion	Laser fluorescence (DIAGNOdent)	Stepwise or selective removal	High diagnostic accuracy, minimizes pulp exposure
Ambiguous results	Combine CDD + DIAGNOdent	Conservative selective removal	Increases diagnostic precision
Real-time intraoperative guidance	FACE system	Fluorescence-assisted removal	Identifies infented dentin via red-orange fluorescence
Final verification	CDD or DIAGNOdent	Confirm absence of carious dentin	Avoid overexcavation

### 4.1 Clinical implications and recommendations

The findings of this review suggest that laser fluorescence devices, particularly DIAGNOdent, provide greater diagnostic accuracy and specificity compared to caries detector dyes, especially in the detection of residual caries in deep lesions. For clinical decision-making, practitioners are advised to use laser fluorescence as a complementary tool to visual-tactile inspection in cases where precise lesion detection is critical, such as near the pulp. Caries detector dyes may still be beneficial in general practice due to their low cost and ease of use but should be applied with caution to avoid over-preparation. When available, combining diagnostic tools with magnification (e.g., loupes or microscopes) can enhance the clinician's ability to differentiate between infected and affected dentin. Selecting the appropriate technique should be based on the clinical scenario, lesion depth, available technology, and the practitioner's training. These insights support an evidence-based, minimally invasive approach tailored to individual patient needs.

Based on this review, we propose a standardized protocol for selective caries removal using the diagnostic systems analyzed. It offers guidance on tool selection—caries detector dyes, DIAGNOdent, and FACE—according to lesion depth and clinical conditions.

### 4.2 Limitations

This scoping review presents several limitations. First, the scope of included studies may limit the statistical robustness and broader applicability of the finding. Second, there is considerable methodological heterogeneity among the included studies in terms of diagnostic criteria, outcome measures, and clinical protocols, which complicates direct comparison. Third, a moderate risk of bias was observed in multiple studies, potentially affecting the reliability of reported outcomes. These limitations highlight the need for future high-quality, standardized clinical trials to validate and expand on these findings. Nonetheless, these limitations do not undermine the current findings but rather highlight opportunities for future, more robust and standardized research.

## 5 Conclusions

1. This scoping review concludes that laser fluorescence systems provide greater diagnostic accuracy than caries detector dyes during selective caries removal. Their higher specificity and sensitivity support improved tissue preservation and reduced risk of pulp exposure. While dyes remain useful, their tendency to overstain may lead to overtreatment. Further research is needed to validate these findings and standardize clinical protocols that integrate fluorescence technologies into minimally invasive caries management. Standardizing diagnostic strategies that combine these tools could enhance decision-making and promote predictable, conservative care in clinical practice.
2. Current evidence on these diagnostic techniques remains limited, primarily due to the small number of studies and the absence of standardized, long-term clinical follow-ups. Further high-quality research including randomized controlled trials with consistent methodologies and rigorous statistical analyses is needed to robustly validate their clinical effectiveness and long-term success in preserving pulp vitality.
